# Supplementary material for: Performance of the Verily Study Watch for measuring sleep compared to polysomnography
Source: Front Sleep. 2024 Dec 13;3:1481878. doi: 10.3389/frsle.2024.1481878 (PMC12713921; doi:10.3389/frsle.2024.1481878)
Supplement: Supplementary file 1 [file Data_Sheet_1.DOCX]

# **Supplementary Material**

**Performance of the Verily Study Watch for Measuring Sleep Compared to Polysomnography**

**Authors**: Sohrab Saeb, PhD; Benjamin W. Nelson, PhD; Poulami Barman, MS; Nishant Verma, PhD; Hannah Allen, BS; Massimiliano de Zambotti, PhD; Fiona C. Baker, PhD; Nicole Arra, BA; Niranjan Sridhar, PhD; Shannon S. Sullivan, MD, MSc; Scooter Plowman, MD, MBA, MHSA, MSc; Erin Rainaldi, MS; Ritu Kapur, PhD; Sooyoon Shin, PhD

Supplementary Table 1. Definitions of overnight sleep measures. These definitions are based on an existing standardization framework (Menghini et al. 2021).

| **Metric** | **Definition** |
| --- | --- |
| **TST** | Total sleep time; total time in minutes classified as sleep (Deep, Light, or REM) between lights-off and lights-on |
| **WASO** | Wake after sleep onset; total time in minutes classified as awake after the first sleep epoch |
| **SE** | Sleep efficiency, defined as percentage of TST over the lights-off to lights-on period |
| **SOL** | Sleep onset latency; total time in minutes classified as wake before the first epoch classified as sleep |
| **NAWK** | Number of awakenings; number of awake periods after the first sleep epoch |
| **Sleep Stage Duration** | Total time in minutes classified as each of the sleep stages (Deep, Light, or REM) between lights-off and lights-on |
| NAWK: Night Awakenings; REM: Rapid Eye Movement; SE: Sleep Efficiency; SOL: Sleep Onset Latency; TST: Total Sleep Time; WASO: Wake After Sleep Onset; REM=Rapid Eye Movement | |

Supplementary Table 2. Participant Characteristics

|  |  | **N=41** |
| --- | --- | --- |
| **Age (years)** | Median (range) | 34.0 (18.0 - 78.0) |
|  | Mean (SD) | 40.5 (16.5) |
| **Age categories,  n (%)** | 18-40 | 25 (61.0) |
|  | 41-80 | 16 (39.02) |
| **Sex, n (%)** | Female | 23 (56.1) |
|  | Male | 18 (43.9) |
| **BMI categories,  n (%)** | < 25 | 30 (73.2) |
|  | ≥ 25 | 11 (26.8) |
| **Skin tone, n (%)** | Light Skin Tone | 21 (51.2) |
|  | Medium Skin Tone | 15 (36.6) |
|  | Dark Skin Tone | 5 (12.2) |
| **Arm hair index,  n (%)** | 1: Little to no visible arm hair, light in color | 17 (41.5) |
|  | 2: Visible, fine, arm hair, light to medium color | 16 (39.0) |
|  | 3 and 4: Coarse and very coarse arm hair, medium to dark color | 8 (19.51) |
| **Race, n (%)** | American Indian or Alaska Native | 1 (2.4) |
|  | Asian | 8 (19.5) |
|  | Black of African American | 4 (9.8) |
|  | Mixed race | 4 (9.8) |
|  | Native Hawaiian or Other Pacific Islander | 1 (2.4) |
|  | Other | 1 (2.4) |
|  | White | 22 (53.7) |
| **Ethnicity, n(%)** | Hispanic or Latino | 4 (9.8) |
|  | Not Hispanic or Latino | 37 (90.2) |
| **Dominant hand,  n (%)** | Ambidextrous | 1 (2.4) |
|  | Left | 5 (12.2) |
|  | Right | 35 (85.4) |
| **OSA score** | Median (range) | 0.0 (0.0 - 7.0) |
|  | Mean (SD) | 1.3 (1.6) |
| **ISI score** | Median (range) | 3.0 (0.0 - 7.0) |
|  | Mean (SD) | 3.0 (1.9) |
| **ESS score** | Median (range) | 5.0 (0.0 - 9.0) |
|  | Mean (SD) | 5.0 (2.7) |
| **AHI Index** | Median (range) | 1.3 (0.1 - 4.7) |
|  | Mean (SD) | 1.7 (1.2) |
| AHI=Apnea hypopnea index ; BMI=body mass index; ESS=Epworth sleepiness scale ; ISI=insomnia severity index; OSA=Obstructive Sleep Apnea; SD=standard deviation | | |

Supplementary Table 3. Confusion matrix for the epoch-by-epoch classification of sleep stages.

|  |  | **Device (VSW)** | | | |  |
| --- | --- | --- | --- | --- | --- | --- |
|  |  | **Wake** | **Light** | **Deep** | **REM** | **Total Reference** |
| **Reference**  **(PSG)** | **Wake** | 5,029 | 869 | 84 | 97 | 6,079 |
|  | **Light** | 1,664 | 15,996 | 1,124 | 1,107 | 19,891 |
|  | **Deep** | 65 | 1,599 | 3,840 | 34 | 5,538 |
|  | **REM** | 470 | 1,269 | 23 | 5,526 | 7,288 |
| **Total Device** | | 7,228 | 19,733 | 5,071 | 6,764 | 38,796 |
| PSG: Polysomnography; REM: Rapid Eye Movement; VSW: Verily Study Watch | | | | | | |

Supplementary Table 4. Performance of ‘sleep vs wake’ classification for participant subgroups.

| **Subgroup (n)** | | **n** | **Sensitivity (95% CI)** | **Specificity (95% CI)** | **NPV (95% CI)** | **PPV (95% CI)** |
| --- | --- | --- | --- | --- | --- | --- |
| **Age** | **18-40 yrs** | 25 | 0.97 (0.97, 0.98) | 0.74 (0.68, 0.79) | 0.85 (0.80, 0.89) | 0.95 (0.94, 0.96) |
|  | **> 40 yrs** | 16 | 0.96 (0.94, 0.98) | 0.65 (0.60, 0.71) | 0.80 (0.71,0.90) | 0.91 (0.88, 0.94) |
| **Sex** | **Female** | 23 | 0.96 (0.95, 0.98) | 0.71 (0.65, 0.76) | 0.78 (0.70, 0.86) | 0.94 (0.92, 0.96) |
|  | **Male** | 18 | 0.98 (0.97, 0.98) | 0.68 (0.62, 0.74) | 0.89 (0.84, 0.92) | 0.92 (0.90, 0.94) |
| **BMI** | **< 25** | 30 | 0.97 (0.96, 0.98) | 0.70 (0.65, 0.75) | 0.83 (0.77, 0.90) | 0.93 (0.91, 0.95) |
|  | **≥ 25** | 11 | 0.97 (0.96, 0.98) | 0.68 (0.61, 0.75) | 0.81 (0.75, 0.88) | 0.94 (0.92, 0.96) |
| **Skin Tone** | **I, II, III** | 21 | 0.98 (0.97, 0.98) | 0.69 (0.65, 0.72) | 0.86 (0.82, 0.89) | 0.93 (0.92, 0.95) |
|  | **IV, V, VI** | 20 | 0.94 (0.91, 0.98) | 0.73 (0.62, 0.92) | 0.75 (0.63, 0.90) | 0.93 (0.89, 0.98) |
| **Arm Hair Index** | **1** | 17 | 0.96 (0.94, 0.98) | 0.69 (0.63, 0.75) | 0.78 (0.68, 0.88) | 0.94 (0.93, 0.96) |
|  | **2** | 16 | 0.97 (0.96, 0.98) | 0.73 (0.66, 0.82) | 0.85 (0.79, 0.90) | 0.94 (0.92, 0.97) |
|  | **3, 4** | 8^a^ | NA | NA | NA | NA |
| **^a^**Due to insufficient number of samples (8) for this subgroup, we did not evaluate the performance BMI = body mass index; CI= confidence interval; NPV = negative predictive value; PPV = positive predictive value | | | | | | |

Supplementary Table 5. Performance of 4-class sleep stage classification for participant subgroups (BMI: body mass index; CI: confidence interval).

| **Subgroup (n)** | | **n** | **Overall Cohen’s Kappa (95% CI)** | **Light Sleep Kappa (95% CI)** | **Deep Sleep Kappa (95% CI)** | **REM Kappa (95% CI)** | **Wake Kappa (95% CI)** |
| --- | --- | --- | --- | --- | --- | --- | --- |
| **Age** | **18-40 yrs** | 25 | 0.70  (0.69, 0.71) | 0.63  (0.42, 0.78) | 0.70  (0.37, 0.90) | 0.74  (0.53, 0.87) | 0.72  (0.50, 0.90) |
|  | **> 40 yrs** | 16 | 0.63 (0.62, 0.64) | 0.56  (0.24, 0.73) | 0.60  (0.12, 0.9) | 0.73  (0.33, 0.9) | 0.66  (0.40, 0.85) |
| **Sex** | **Female** | 23 | 0.66  (0.65, 0.67) | 0.59  (0.26, 0.75) | 0.66  (0.23, 0.87) | 0.73  (0.35, 0.90) | 0.70  (0.41, 0.89) |
|  | **Male** | 18 | 0.68  (0.67, 0.69) | 0.61  (0.42, 0.78) | 0.66  (0.26, 0.92) | 0.74  (0.53, 0.89) | 0.69  (0.50, 0.88) |
| **BMI** | **< 25** | 30 | 0.67  (0.67, 0.68) | 0.6 (0.27, 0.78) | 0.67  (0.24, 0.91) | 0.73  (0.34, 0.90) | 0.70  (0.42, 0.90) |
|  | **≥ 25** | 11 | 0.67  (0.65, 0.68) | 0.59  (0.43, 0.73) | 0.63  (0.26, 0.87) | 0.74  (0.65, 0.84) | 0.68  (0.53, 0.82) |
| **Skin Tone** | **I, II, III** | 21 | 0.70  (0.69, 0.71) | 0.62  (0.45, 0.78) | 0.67  (0.28, 0.92) | 0.77  (0.61, 0.90) | 0.71  (0.52, 0.85) |
|  | **IV, V, VI** | 20 | 0.64  (0.63, 0.65) | 0.57  (0.24, 0.74) | 0.65  (0.20, 0.87) | 0.70  (0.30, 0.88) | 0.68  (0.40, 0.90) |
| **Arm Hair Index** | **1** | 17 | 0.65  (0.64, 0.66) | 0.59  (0.28, 0.78) | 0.62  (0.19, 0.88) | 0.74  (0.38, 0.89) | 0.69  (0.41, 0.84) |
|  | **2** | 16 | 0.689  (0.67, 0.69) | 0.60  (0.37, 0.75) | 0.69  (0.33, 0.87) | 0.73  (0.49, 0.89) | 0.72  (0.51, 0.91) |
|  | **3,4** | 8^a^ | NA | NA | NA | NA | NA |
| **^a^**Due to insufficient number of samples (8) for this subgroup, we did not evaluate the performance BMI = body mass index; CI= confidence interval | | | | | | | |
